# Organic Passivation of Deep Defects in Cu(In,Ga)Se_2_ Film for Geometry-Simplified Compound Solar Cells

**DOI:** 10.34133/research.0084

**Published:** 2023-03-29

**Authors:** Jingwei Chen, Xuan Chang, Jianxin Guo, Qing Gao, Xuning Zhang, Chenxu Liu, Xueliang Yang, Xin Zhou, Bingbing Chen, Feng Li, Jianming Wang, Xiaobing Yan, Dengyuan Song, Han Li, Benjamin S. Flavel, Shufang Wang, Jianhui Chen

**Affiliations:** ^1^ Advanced Passivation Technology Lab, College of Physics Science and Technology, Hebei University, Baoding, 071002, China.; ^2^ State Key Laboratory of Photovoltaic Materials & Technology, Yingli Green Energy Holding Co., Ltd., Baoding 071051, China.; ^3^ Das Solar Co., Ltd., No 43 Bailing South Road, Quzhou Green Industry Clustering Zone, Quzhou, Zhejiang Province, 324022, China.; ^4^ Institute of Nanotechnology, Karlsruhe Institute of Technology, Hermann-von-Helmholtz-Platz 1, Eggenstein-Leopoldshafen, 76344, Germany.

## Abstract

Diverse defects in copper indium gallium diselenide solar cells cause nonradiative recombination losses and impair device performance. Here, an organic passivation scheme for surface and grain boundary defects is reported, which employs an organic passivation agent to infiltrate the copper indium gallium diselenide thin films. A transparent conductive passivating (TCP) film is then developed by incorporating metal nanowires into the organic polymer and used in solar cells. The TCP films have a transmittance of more than 90% in the visible and nearinfrared spectra and a sheet resistance of ~10.5 Ω/sq. This leads to improvements in the open-circuit voltage and the efficiency of the organic passivated solar cells compared with control cells and paves the way for novel approaches to copper indium gallium diselenide defect passivation and possibly other compound solar cells.

## Introduction

Nonradiative recombination via defects in photovoltaic (PV) materials and at their interfaces is the main source of performance loss for solar cells [1–7]. Passivation technologies are employed to suppress nonradiative carrier recombination and have thus become key to the enhancement of PV devices from silicon, CuIn_1−*x*_Ga*_x_*Se_2_ (CIGS), and perovskites. The passivation of defects in silicon with thin dielectric films such as atomic-layer-deposited (ALD) Al_2_O_3_, plasma-enhanced chemical-vapor-deposited SiN*_x_*, and high-temperature-formed SiO_2_, has been used for ~40 years [8,9] and have helped silicon solar cells become the mainstream technology in the modern PV industry. These solar cells currently reach power conversion efficiencies (*PCE*s) of up to 26.7% [10]. Even for the comparably younger perovskite solar cells, in recent years the passivation of bulk and interface defects [11,12] has led to *PCEs* over 25% [13–16]. Combining with other optimized material strategies and device designs, silicon and perovskite solar cells have achieved 91% and 87% of the Shockley–Queisser (SQ) limit power conversion efficiency (*PCE*) (29.4%), respectively [17].

In contrast, CIGS solar cells have only reached 79.4% of the possible SQ limit and the highest reported *PCE* is 23.35% [18] by far, and thus, there are still many improved spaces. The open-circuit voltage (*V*_OC_) and the fill factor (*FF*) of the CIGS solar cells are limited by recombination-active defect states at interfaces as well as in the bulk [19–21]. These defect states are mainly generated by unterminated bonds, such as indium copper antisite In_Cu_, selenium vacancy V_Se_, and gallium copper antisite Ga_Cu_. They exist on the outer surface, at inner grain boundaries, and at lattice points in the CIGS material [22,23]. Because of the diverse nature of the possible defects, the development of an effective passivation technology capable of treating all types simultaneous is a challenge. In this direction, alkali elements are usually added into the CIGS material and have been shown to afford high *PCE*s. Na and K have been shown to effectively passivate defects (In_Cu_ and Ga_Cu_) and K, Rb, and Cs help to form a Cu-poor surface layer and an inversion layer on CIGS surface. For example, Chirilă et al. [40] achieved the record efficiency 20.4% in 2013 on PI (alkali-free) substrate by alkali (Na and K) postdeposition treatment (PDT). Since then, further breakthroughs in CIGS solar cell efficiency have always been accompanied by a PDT technology. In 2016, Jackson et al. [24] demonstrated the effect of the rubidium and caesium in the alkali PDT as applied to CIGS solar cell absorbers and reported 22.6% efficiency. In 2019, Nakamura et al. [18] combined CsF-PDT and Cd-free double buffer layer based on S-incorporated Cu(In,Ga)–(Se,S)_2_ (CIGSSe) thin film. As a result, the efficiency was improved to 23.35%. These indicate the key role of defects passivation in improving CIGS solar cell efficiency.

Still, active research needs to be undertaken to further enhance passivation of the CIGS interfaces and grain boundaries. For example, very recently, inspired by passivated emitter rear contact silicon solar cells, thin dielectric films such as Al_2_O_3_, HfO_2_, and GaO*_x_*, have already been widely investigated [26–29]. However, note that the atomic-layer-deposited Al_2_O_3_ can only passivate the outermost defects and is not able to extend to the grain boundary defects [27]. In addition, as with silicon PVs [30,31], the use of high-temperature processes or high-vacuum equipment brings about technological complexities for device fabrication as well as the corresponded employment of tedious materials such as intrinsic ZnO (i-ZnO) and aluminium-doped ZnO (AZO) or ITO, and even recently developed Zn(O,S)/(Zn,Mg)O stack layer. However, these materials cannot passivate defects and provide higher light incoming, which limits simplification of device structure design [32–34].

Here, we present an organic passivation method, using polystyrene sulfonic acid (PSS) or Nafion to efficiently reduce the CIGS interface recombination. This passivation technology extends its passivation effect to grain boundary defects by the infiltration of organic solution into the CIGS thin films. This simple and vacuum-free approach can be performed at room temperature, and most importantly, it is suitable for polycrystalline compound thin films with a high density of grain boundaries. On the basis of this passivation method, we then develop a new organic passivated CIGS solar cell, with a simplified architecture. Transparent conductive passivating (TCP) films are produced by integrating silver nanowires (AgNWs) into the PSS or Nafion polymer matrix to form composite films, referred to as AgNWs:PSS or AgNWs:Nafion herein, which combined with their excellent passivation effect, we can build the architecture I: TCP/i-ZnO/CdS/CIGS/Mo solar cell and the simpler architecture II: TCP/CdS/CIGS/Mo solar cell without the conventional transparent conductive oxides (TCOs) such as ITO or AZO or even the i-ZnO in CIGS cells. We show that the organic passivated CIGS solar cells marked outperform unpassivated control cells in *PCE*, which increases from 12.27% to 16.87% on the architecture I by addition of Nafion passivation and from 8.91% to 13.48% on the architecture II by addition of PSS passivation.

## Results

### Organic passivation of CIGS thin films

**Fig. 1.**
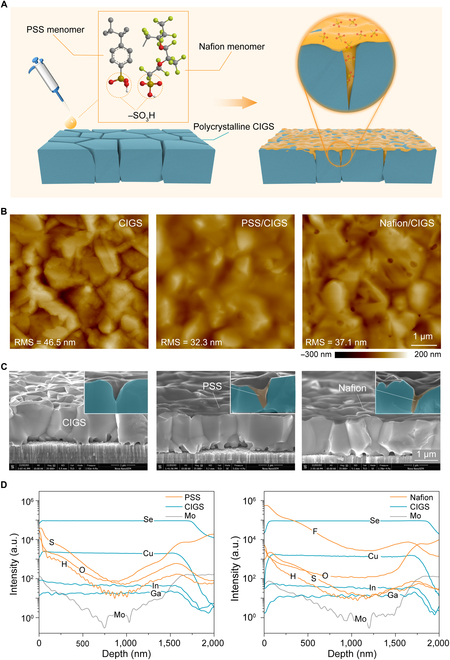
Organic passivation of CIGS thin films. (A) Schematic of the organic PSS and Nafion-passivated CIGS thin film. (B) Pristine CIGS, PSS/CIGS, and Nafion/CIGS samples as measured by AFM. (C) SEM cross-sectional image of pristine CIGS, PSS/CIGS, and Nafion/CIGS samples. (D) Depth profiles in SIMS of PSS/CIGS and Nafion/CIGS samples. RMS, root mean square.

CIGS thin films with a thickness of approximately 1.6 μm are deposited by co-evaporation on Mo-coated soda-lime glass in a standard 3-stage process [35]. Figure 1A shows the schematic illustration of the organic PSS and Nafion-passivated CIGS thin film. Both PSS and Nafion contain the –SO_3_H functional group, which has been shown to afford a high-quality passivation effect on silicon surface defects [36,37]. Here, we extend this technology onto CIGS materials and solar cells and further demonstrate its efficacy for compound semiconductor defects. When the precursor solutions of PSS or Nafion are drop-casted on the CIGS surface, an organic layer is formed on the outer surface of the CIGS (referred to as PSS/CIGS and Nafion/CIGS herein), and the inner pits are filled by solution infiltration. As shown in the Atomic Force Microscope (AFM) images in Fig. 1B, the PSS or Nafion smooths the CIGS surface and decreases root-mean-square roughness. Figure 1C shows a Scanning Electron Microscope (SEM) image of the CIGS, PSS/CIGS, and Nafion/CIGS, where excellent surface coverage by PSS and Nafion and organic infiltration into the CIGS pits can be seen. The ability to infiltrate the pits of CIGS was further verified by performing a depth profile in secondary ion mass spectrometry (SIMS). For the compositional analysis of the samples, Figure 1D shows graded distributions of the PSS and Nafion from top to middle to bottom of the CIGS layer. The depth profiles of S, F, H, and O through the CIGS suggested that PSS and Nafion can infiltrate the CIGS layer and accumulate at the front surface and back interface. This is similar to the alkali-PDT method reported previously [38–42]. The accumulation of elements at the back interface is related to the penetrated passivation layer at the Mo interface through the large number of voids at the backside (Fig. 1C). Those voids are comment for the CIGS processes, especially for the electrodeposition method [43]; more easily formed voids allow us to expect the better passivation effect for electrodeposited CIGS solar cells. Note that high concentration of Mo at front interface is a measurement artifact that may be caused by the impurities with the same molecular weight with Mo, which can also be found elsewhere [44,45].

**Fig. 2.**
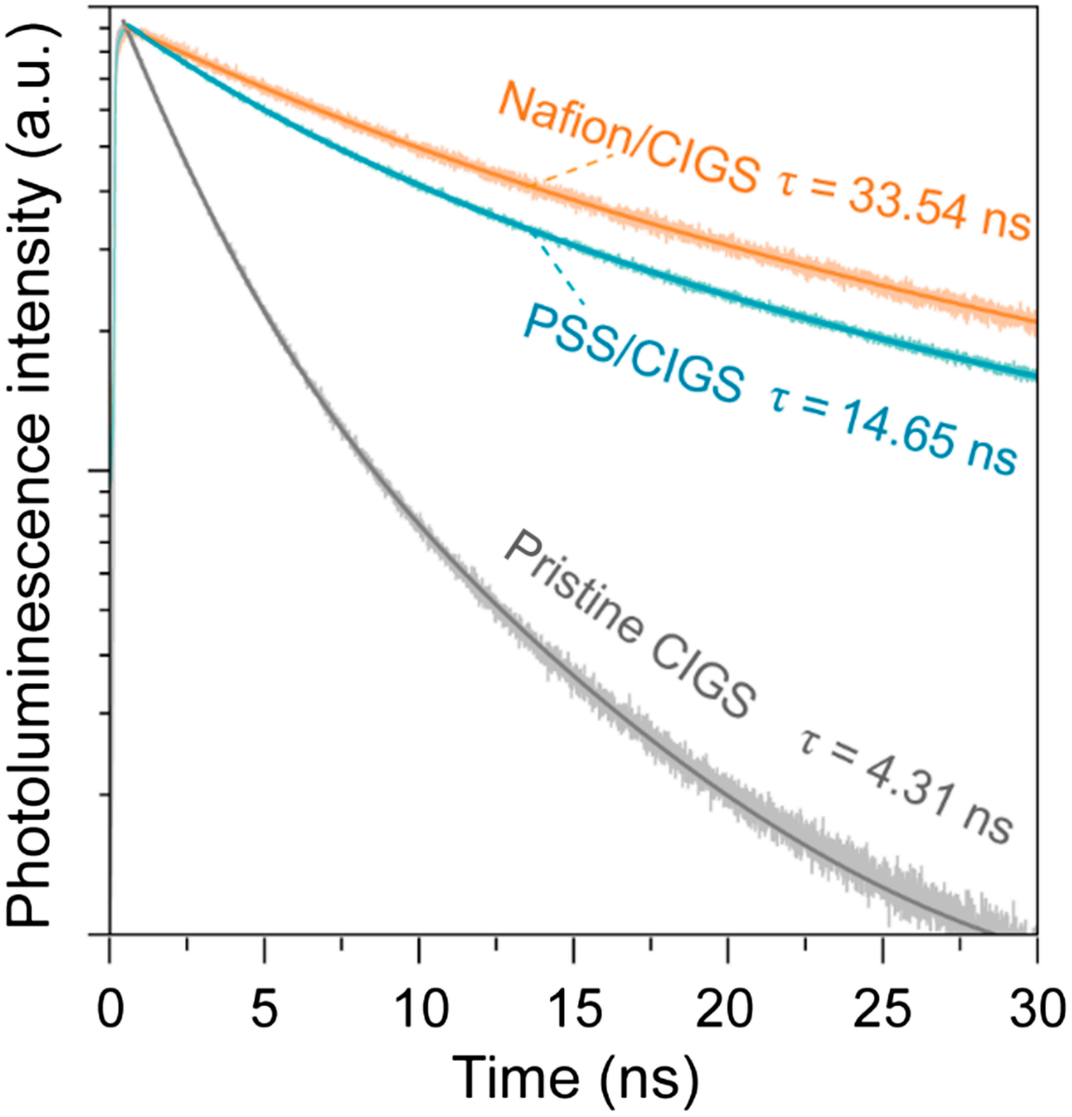
Organic passivation improves minority carrier lifetime. TRPL decay curve of pristine CIGS, PSS/CIGS, and Nafion/CIGS.

**Fig. 3.**
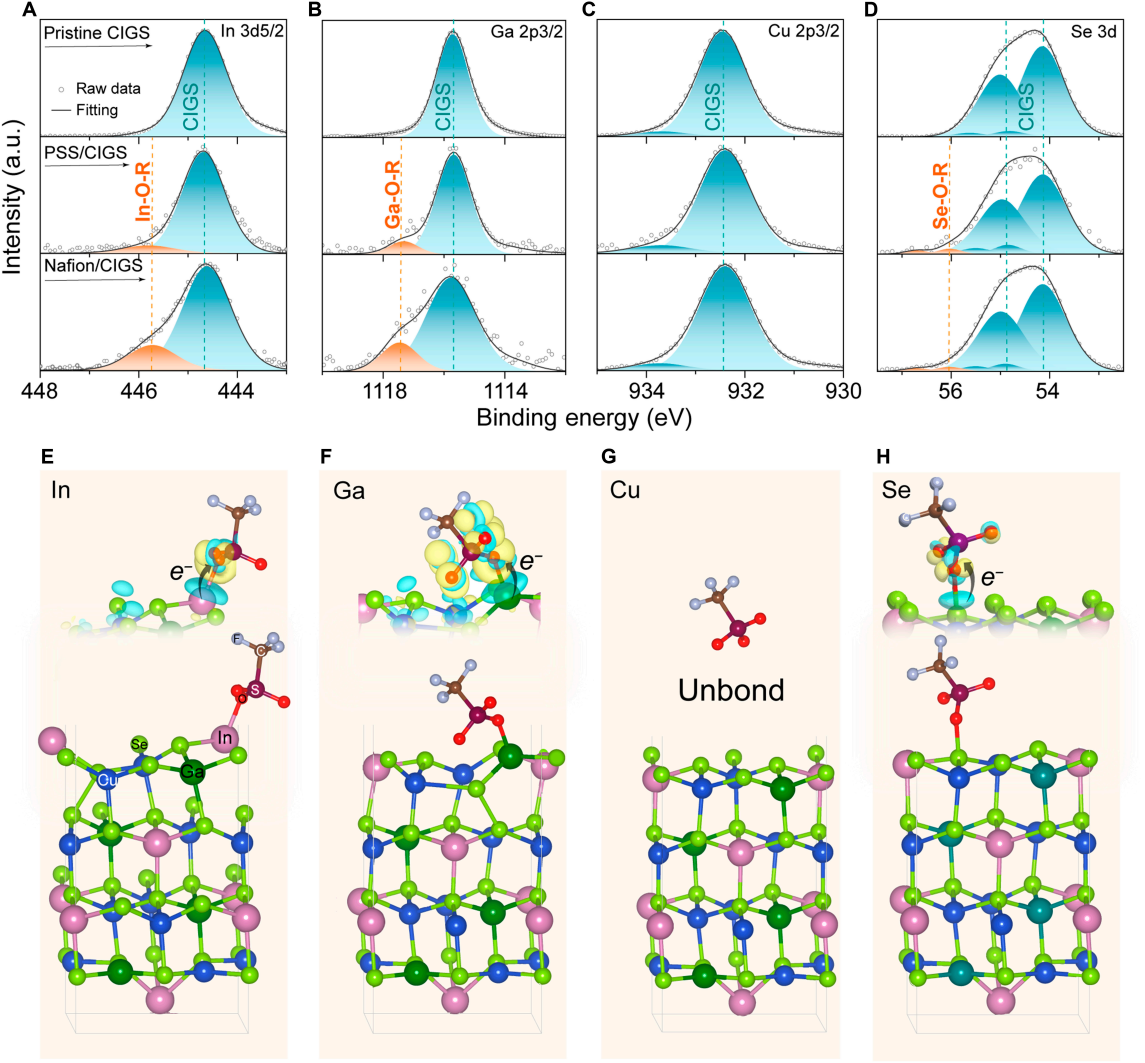
The reaction between sulfonate and CIGS surface and theoretical calculation. (A to D) XPS core level spectra of (A) In 3d5/2, (B) Ga 2p3/2, (C) Cu 2p3/2, and (D) Se 3d of CIGS, PSS/CIGS, and Nafion/CIGS samples. (E to H) Nafion adsorption on the CIGS (112) surface with different atoms. (E) In, (F) Ga, (G) Cu, and (H) Se. The inset images are the corresponding charge density different for O with different surface atoms expect Cu. Yellow and green isosurfaces represent charge accumulation and depletion regions, respectively.

Time-resolved photoluminescence (TRPL) measurements were performed to prove the passivation effect. The carrier lifetime is calculated by a common biexponential kinetic model [46,47]. As shown in Fig. 2, the initially low effective lifetime of 4.31 ns increases to 14.65 and 33.54 ns, after PSS and Nafion passivation, respectively. Such improvements in carrier lifetime allow us to roughly extract the quasi Fermi level splitting (*qFLs*) of the passivated CIGS absorber by using the method (Eq. 1) recently reported by Siebentritt et al. [48–50],

$$\Delta \left(\Delta E_{F}\right)=k_{B}T\text{ln}\left(\frac{\tau_{1}}{\tau_{2}}\right)$$ (1)

where ∆(∆*E_F_*) is the *qFLs*, *k_B_* is the Boltzmann’s constant, *T* is the temperature, and *τ_1_* and *τ_2_* are the carrier lifetime of the passivated and unpassivated CIGS, respectively. An increase of 31.8 and 53.3 meV in *qFLs* are obtained for the PSS and Nafion passivated CIGS, indicating the improvement in *V*_OC_ of the further CIGS solar cell devices. X-ray photoelectron spectroscopy (XPS) was employed to clarify the origin of passivation. Ar^+^ etching technology was added to remove the surface native oxides or top organic covering layer so that the characteristic matches the probe range (5 to 10 nm) of XPS [51]. Fig. 3A to D shows XPS spectra of In 3d5/2, Ga 2p3/2, Cu 2p3/2, and Se 3d of CIGS, PSS/CIGS, and Nafion/CIGS samples. From the In and Ga XPS spectra, aside from In and Ga species in the CIGS main component, In and Ga oxides are detected (Fig. 3A and B), and this suggests that the grafting of O in the sulfonic functional group in the PSS or Nafion molecule onto the CIGS surface defects has occurred. In this case, acceptor defects such as In_Cu_ and Ga_Cu_ at CIGS interface and ground boundaries will be passivated. and Cu 2p XPS spectra show no obvious change and remain stable before and after PSS or Nafion treatment (Fig. 3C). For Se, the organic passivation leads to the appearance of Se suboxides (Se^*x*+^, 0<*x*<2) identified toward higher binding energy (Fig. 3D), which again indicate that surface Se defects have been passivated by –SO_3_H [51].

First-principles calculations were employed to further clarify physical details of passivation. A simple trifluoromethane sulfonic acid molecule was used to represent the –SO_3_H based material, and further details on the computational methods can be found in the supporting information. By performing the structure optimization and the charge density difference analysis, 3 adsorption structures for the –SO_3_H group onto CIGS (112) surface are demonstrated (Fig. 3E to H). With the surface reconstructure of CIGS (112) surface, the –SO_3_H group can bond with Ga, In, and Se by its O atom, the corresponding adsorption energies are −1.80, −1.75, and −1.47 eV, respectively, which suggest that 3 states are stable. However, for the Cu, no stable adsorption structures are obtained, and therefore, the –SO_3_H group cannot passivate Cu defects. In the insets to Fig. 3E, F, and H, it can be seen that the formation of the In–O, Ga–O, and Se–O bond is accompanied by a redistribution of surface electrons to the organic-inorganic interface and the formation of a depletion region at CIGS surfaces. These 3 theoretical calculated geometries are in good agreement with the XPS measurements and lead us to present the following passivation scheme in which In_Cu_ and Ga_Cu_ are oxidized by the –SO_3_H according to the following process:

$${\left[\text{In},\mathrm{Ga}\right]}_{\left(\mathrm{Cu},\text{surf}.\right)}+X-{\mathrm{SO}}_{3}H\to {\left[\text{In},\mathrm{Ga}\right]}_{\left(\mathrm{Cu},\text{surf}.\right)}-O-R$$ (2)

and,

$${\left[\mathrm{Se}\right]}_{\left(\mathrm{DB}\right)}+X-{\mathrm{SO}}_{3}H\to \mathrm{Se}-O-R$$ (3)

where X represents a surplus constituent other than a sulfonic functional group, –SO_3_H and R is the surplus O-containing constituent in the X–SO_3_H molecule. In this case, those defect states, such as In_Cu_, V_Se_, and Ga_Cu_, can be effectively passivated by organic functional group termination method.

### TCP films

**Fig. 4.**
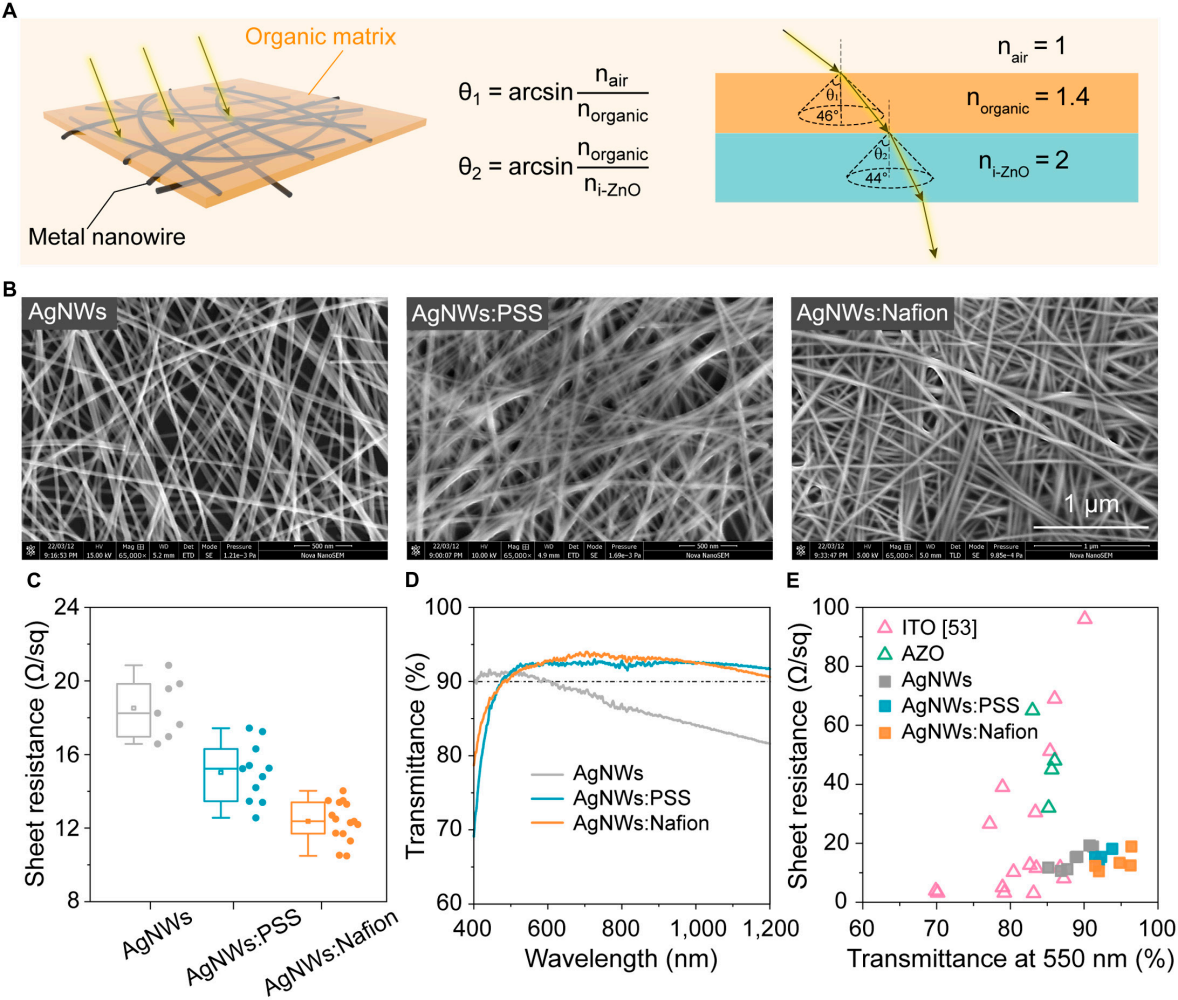
Transparent conductive passivating (TCP) films. (A) Schematic illustration of AgNWs:PSS or Nafion organic cohesive metal nanowire network and schematic diagram of the optical path for Nafion or PSS to realize antireflection. (B) SEM image of AgNWs, AgNWs:PSS, and AgNWs:Nafion composite transparent conductive film. (C) Sheet resistances of AgNWs, AgNWs:PSS, and AgNWs:Nafion films. (D) Spectral transmittance of AgNWs, AgNWs:PSS, and AgNWs:Nafion films. (E) Comprehensive comparison of optical and electrical properties of TCO materials films (ITO and AZO) and AgNWs, AgNWs:PSS, and AgNWs:Nafion films

In addition to excellent passivation of the CIGS thin films, PSS or Nafion can also be used as a transparent conductive electrode when combined with a thin film of AgNWs. Because of lower refractive index of PSS or Nafion compared to TCO-based materials [52], this organic/metal nanowire will theoretically possesses higher transmittance (Fig. 4A). SEM images in Fig. 4B show that the coverage of the PSS or Nafion on the top of the AgNWs networks merges nanowires and thus enhances conductivity of thin films (Fig. 4C). Note that because of the porosity of AgNWs, the PSS or Nafion incorporated into the AgNWs networks, it is actually a composite film, i.e., AgNWs:PSS or AgNWs:Nafion. Figure 4D shows the transmittance of the controlled AgNWs, AgNWs:PSS, and AgNWs:Nafion films, and an obvious enhancement in transmittances can be seen on the composite thin films. Most notably, the incorporation of Nafion enhanced the transmittance of the AgNWs films up to >90.6% in the wavelength range 400 to 1,200 nm. It also lead to a sheet resistance of only 10.5 Ω/sq. This is better than the previous transparent conductive electrode materials as compared in Fig. 4E [53]. As such, our approach achieves the best trade-off between optical and electrical properties among current transparent conductive materials (Fig. S1). The ability of these materials to passivate defects in devices such as CIGS solar cells leads us to coin the term “Transparent conductive passivating (TCP) films”, and it is predicted that these may be able to be used in a window or contact layer in other photoelectric devices.

### Device design and characterization

**Fig. 5.**
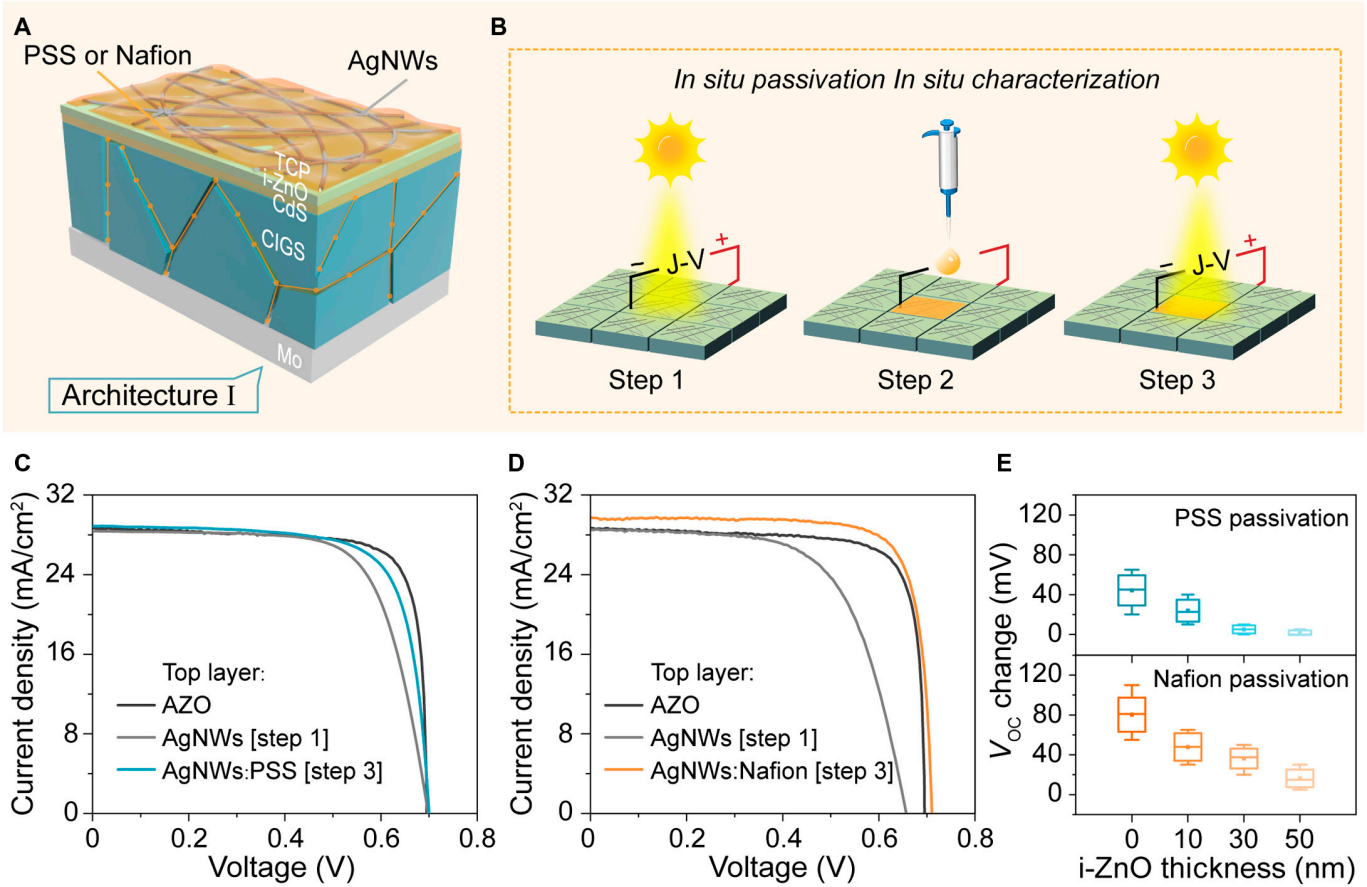
Device and characterisation of architecture I. (A) Structural diagram of the solar cell of architecture I (TCP/i-ZnO/CdS/CIGS/Mo). (B) Schematic illustration of in situ passivation and in situ characterization (Step 1: measuring the current density–voltage (*J-V*) characteristic of AgNWs/i-ZnO/CdS/CIGS/Mo solar cell; Step 2: adding organic passivation agent PSS or Nafion and forming TCP on the top of the cell without any changes of the sample position or probes; Step 3: measuring *J-V* again). (C and D) The comparison of *J-V* curves of the architecture I solar cells before and after adding the PSS or Nafion passivation and using AZO as the window layer were used as a control. (E) Change of *V*_OC_ with i-ZnO thickness.

The TCP film replaces the AZO or ITO layer in CIGS solar cells that do not have a passivation effect. The first solar cell architecture used in depicted in Fig. 5A and consisted of the layers TCP/i-ZnO/CdS/CIGS/Mo. The CdS thickness was ~50 nm and was deposited by chemical bath deposition (CBD), and the i-ZnO layer (10 to 50 nm) was deposited on the top of CdS by radio frequency sputtering. TCP films were then fabricated by spin-coating a AgNWs film and then covering it with an organic passivation agent. In this device design, the TCP film simultaneously acts as a light transmitting, conductive, and antireflective layer but also as the passivation layer for CIGS. Here, the PSS or Nafion liquid can penetrate the i-ZnO/CdS stack layer and reach the CIGS interface and inside through porous AgNWs (Fig. S2) because the i-ZnO is thin and the coverage of CBD-CdS incomplete [54,55]. This is verified by an in situ passivation and in situ characterization shown in Fig. 5B. It involves measuring the current density–voltage (*J*-*V*) characteristic of as-prepared AgNWs/i-ZnO/CdS/CIGS/Mo solar cell (step 1); adding organic passivation agent PSS or Nafion and forming TCP on the top of cell without any changes of the sample position or probes (step 2); and measuring *J*-*V* again (step 3). Figure 5C and D show the *J*-*V* curves of the solar cells before and after adding the PSS or Nafion passivation, respectively, and Table 1 shows their PV performance parameters. As shown, TCP addition yielded a dramatic improvement in all performance parameters including short-circuit current density (*J*_SC_), *V*_OC_, *FF*, and *PCE* compared to the TCP-free controls. The improvement in *J*_SC_ and *FF* can be explained to be the enhanced transmittance and conductivity contributed by TCP films, which is consistent with sheet resistance and transmittance in Fig. 4. The improvement in *V*_OC_ is related to the infiltration passivation of PSS or Nafion, but this effect was found to be dependent upon the thickness of the i-ZnO layer which determined how much solution penetrated to the CIGS absorber. Figure 5E shows that *V*_OC_ decreases with the increase of the i-ZnO thickness and that an effective thickness is <30 and <50 nm for PSS and Nafion passivation, respectively. Between step 1 and step 3 the addition of the TCP layer resulted in a *PCE* increase of13.9% to 15.25% and 12.27% to 16.87%, for the PSS and Nafion, respectively. It can also be seen that the TCP-based cell achieved a higher *PCE* compared to reference AZO-based cells.

**Table 1.** Comparison of PV performance parameters of solar cells of architecture I (TCP/i-ZnO/CdS/CIGS/Mo) with different top layer.

Top layer	*V*_OC_ (mV)	*J*_SC_ (mA/cm^2^)	*FF* (%)	*PCE* (%)
AZO	694.47	28.68	80.25	15.99
AgNWs [step 1]	694.47	28.36	70.55	13.90
AgNWs:PSS [step 3]	699.50	29.35	74.28	15.25
AgNWs [step 1]	669.35	28.55	64.22	12.27
AgNWs:Nafion [step 3]	709.55	29.76	79.90	16.87

**Fig. 6.**
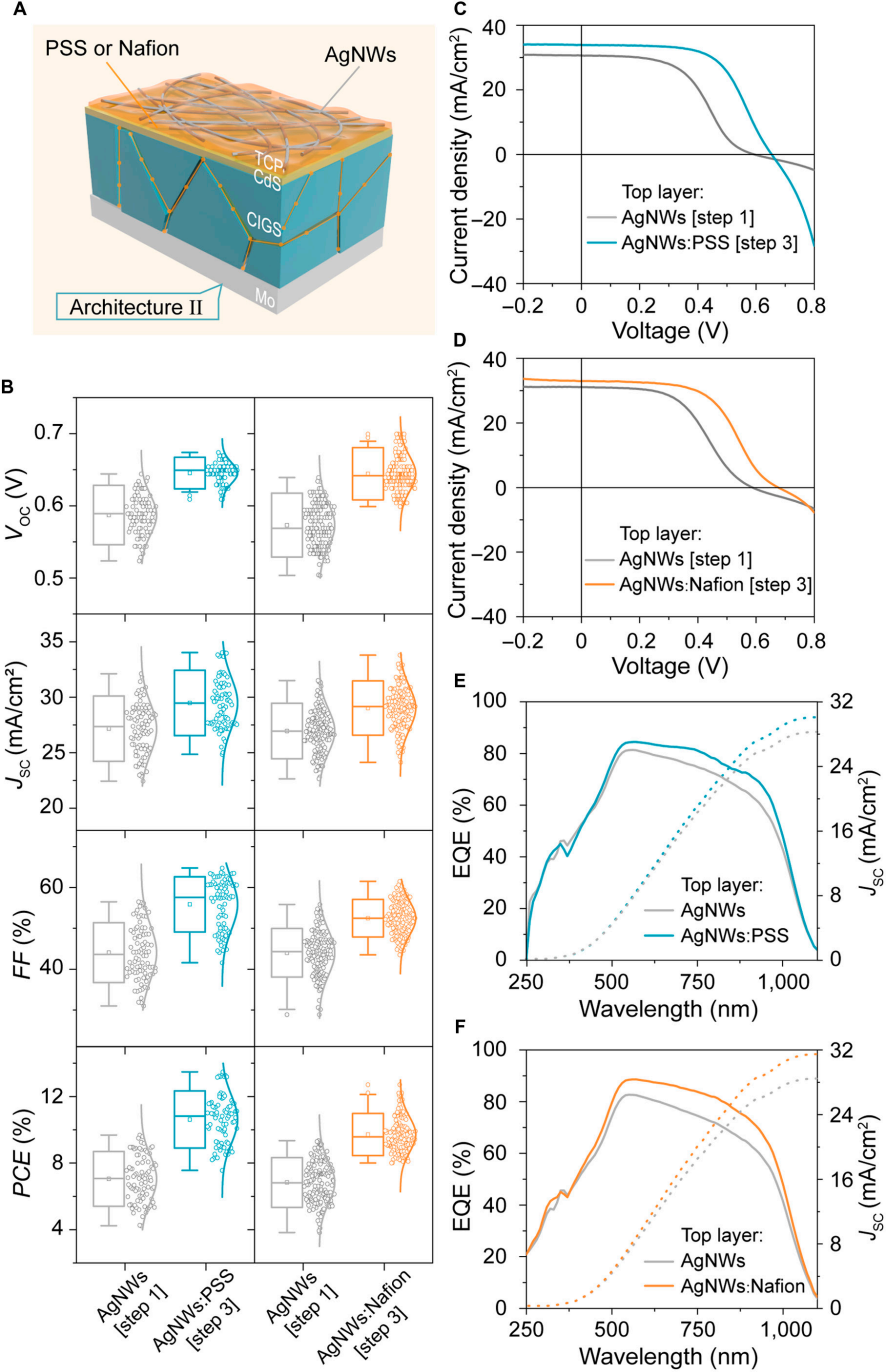
Device and characterization of architecture II. (A) Structural diagram of the solar cell of architecture II (TCP/CdS/CIGS/Mo). (B) Comparison of architecture II solar cells performance (*V*_OC_, *J*_SC_, *FF*, and *PCE*) before and after passivation. (C and D) The comparison of *J-V* curves of the architecture II solar cells before and after adding the PSS or Nafion passivation. (E and F) The comparison of EQE curves of the architecture II solar cells before and after adding the PSS or Nafion passivation.

The excellent optoelectronic properties of the TCP allow us to completely remove the i-ZnO layer and develop the simpler device structure consisting of the layers TCP/CdS/CIGS/Mo solar cell (architecture II) (Fig. 6A). In this case, because of the lack of the i-ZnO barrier, the organic passivation agent is able to easily to infiltrate into CIGS interface. Figure 6B and Table 2 show in situ passivation and in situ characterization of a total of 234 subsolar cells on 21 10 cm × 10 cm CdS/CIGS/Mo cells. Because of more thorough infiltration of PSS or Nafion, compared with the cells in Fig. 5, the *V*_OC_ change now becomes more apparent and also the increase in *J*_SC_, *FF*, and *PCE* is larger. Note that *J*_SC_ in the architecture II is higher than that in the architecture I because the parasitic absorption from the i-ZnO is removed. Figure 6C and D show the *J*-*V* curves of the champion solar cells. It is found that a AgNWs/CdS/CIGS/Mo cell had a S-shaped *J-V* curve, which originated from the reduced electric field and increased recombination losses. However, when the TCP scheme is used, the S-shaped problem is alleviated. The same results can be proved by the TCP films with different concentrations of PSS (Fig. S3). Figure 6E and F show the external quantum efficiency (EQE) curves of the cells; clearly, obvious improvement can be observed and is consistent with the transmittance improvement in Fig. 4C. As there was no transparent oxide conductive layer absorbing short wavelength light, the response of architecture II in short wavelength band was enhanced compared with the control (Fig. S4).

**Table 2.** Comparison of PV performance parameters of solar cells of architecture II (TCP/CdS/CIGS/Mo) with different top layer.

Top layer	*V*_OC_ (mV)	*J*_SC_ (mA/cm^2^)	*FF* (%)	*PCE* (%)
AgNWs [step 1]	588.97	30.61	49.41	8.91
AgNWs:PSS [step 3]	649.33	33.85	61.32	13.48
AgNWs [step 1]	583.94	31.11	49.38	8.97
AgNWs:Nafion [step 3]	674.43	32.97	54.78	12.18

## Discussion

This work opens a new direction for CIGS passivation using organic layers and it is predicted that it may be useful in other compound semiconductor devices such as Cu_2_ZnSnS_4_(CZTS), CdTe, and Sb_2_Se_3_ solar cells. In this work, a 15% *PCE*-level CIGS was taken as the baseline, but with further improvements in the CIGS cell, higher efficiencies are expected. In addition, the use of a TCP film enables simple CIGS solar cell architectures to be used that are also suitable for flexible substrate solar cells. The approach matches perfectly with existing nonvacuum processing of the compound solar cells such as the electrodeposition, solution, or hydrothermal methods and provide a new technological route for thin film solar cells. The long-term stability of the organic passivation has been discussed in the previous work, and the main task is the appropriate encapsulation against the adsorption of water [36,56,57].

In summary, we discovered the organic passivation strategy for CIGS solar cells with the most diversified defects, which achieved the infiltration passivation of both surface and inner ground boundaries of the CIGS. We developed a TCP material, which possess multiple functions, such as over 90.6% high transmittance and only 10.5 Ω/sq sheet resistances simultaneously, and excellent passivation on the CIGS solar cells. New passivation technologies and TCP materials gives birth to 2 kinds of new architectures for CIGS solar cells, for example, an AZO/i-ZnO-free TCP/CdS/CIGS/Mo solar cell, which simplified the CIGS structure and process. Because of the passivation effect of the TCP, *V*_OC_ has a dramatical increase compared with the unpassivated control, and we find that *V*_OC_ increasement is the dependence of the infiltration depth of organic solution, specifically, in the AZO-free TCP/i-ZnO/CdS/CIGS/Mo solar cell, *V*_OC_ change is determined by the i-ZnO thickness. The maximum *V*_OC_ change is the zero thickness i-ZnO, in this case, the cell illustrates the S-shape *J-V* curve, which can be solved by addition of organic passivation. The organic passivation paves the way to further increase the CIGS *PCE*/SQ limit *PCE* ratio via the elimination of diversified defects and is a new point forward simplifying the CIGS cells and obtaining low-cost, highly efficient solar cells.

## Materials and Methods

### Solution and thin-film preparation

The PSS solutions with different concentrations (0.5, 1, 3, and 6 wt.%) were obtained by mixing the PSS (Jixin, 30% original PSS aqueous solution was dried) with ethanol (Sigma-Aldrich, > 99.7 wt.%). Then, simple magnetic stirring for 3 h was used to obtain the uniform precursor solution. The Nafion solutions with different concentrations (0.3, 0.6, 1.7, 3.5, and 5.8 wt.%) were obtained by mixing an original 20-wt.% Nafion (Sigma-Aldrich, 20 wt.% in a mixture of lower aliphatic alcohols and 34% water) with ethanol (> 99.7 wt.%). Again, simple magnetic stirring for 2 h was used to obtain the uniform precursor solution. The AgNWs solutions were diluted from 1-wt.% sliver nanowires ink (Kechuang, AW030-LP, *Ave. D* = 25 to 35 nm, *Ave. L* = 10 to 20 μm, ethanol solution) with ethanol (>99.7 wt.%) to 0.25 wt.%. AgNWs thin films were fabricated on glasses using a spin-coating method at 3,500 rpm to measure transmittance and square resistance, and AgNWs thin films were also deposited on the surface of CdS layers and i-ZnO films as the TCO layer by the same method. Further, the PSS and the nafion solutions were dropped on the surface of AgNWs thin films as passivation films.

### Device fabrication

Three architectures of solar cells were fabricated, including I: TCP/i-ZnO/CdS/CIGS/Mo; II: TCP/CdS/CIGS/Mo; and controlled: AZO/i-ZnO/CdS/CIGS/Mo (Fig. S5). Device fabrication involves several steps: 1) The CIGS films (~1.6 μm) were deposited onto Mo-coated soda-lime glass in a high vacuum chamber for sizes up to 10 × 10 cm^2^ using so-call 3-stage coevaporation process. More details about the coevaporation system could be referred to our previous study [35]. The element ratio of [Cu]/([Ga] +[In]) and [Ga]/([Ga] + [In]) was controlled at 0.88 and 0.38 (atomic percent), respectively. 2) After the CIGS films were fabrication, the CdS thin films (~50 nm) were grown on the CIGS as the buffer layer by CBD at 68 °C for 11 min. 3) Subsequently, the intrinsic zinc oxide (i-ZnO) thin films(~80 nm) /n-type aluminium doped ZnO (ZnO:Al,~300 nm) window layers were deposited by radio frequency magnetron sputtering. 4) Further, uniform films of AgNWs were formed on the surface of CdS layers via spin coating. The organic agent (PSS or Nafion) was dropped on the surface of the AgNWs. The total area of each device is approximately 0.25 cm^2^ defined by mechanical scribing.

### Characterization

The surface morphologies of the CIGS thin films were measured by field emission scanning electronic microscopy (FEI NOVA NANOSEM 450) and atomic force microscopy (Bruker Multimode 8). The surface analysis was performed by XPS (Thermo Fisher Scientific ESCALAB 250Xi) in ultrahigh-vacuum conditions (base pressure of 1 × 10^-9^ mbar) using an Al Kα x-ray source (15kV, 10mA, 500-μm spot size). The energy scale was calibrated using C1s at 284.80eV. The deconvolution and peak fitting were carried out using the software “Avantage”. The optical properties of the the AgNWs, AgNWs:PSS, AgNWs:Nafion films, and ITO transparent conductive glass were performed using the ultraviolet-vis-near-infrared spectrophotometer (Hitachi U4100) with a 60-mm-diameter integrating sphere. The element content depth profiling of the CIGS thin films was measured by time-of-flight SIMS (TOF-SIMS 5-100 system from IONTOF). The analysis beam was 30-keV primary Bi^+^, and the sputtered area was 100 ×100 μm^2^. The sputtering beam was Cs^+^ for the sputter beam with 2 keV and a rasterized area of 220 × 220 μm^2^. PL and TRPL measurement were carried out using a pulsed laser with a repetition rate of 20 MHz at a wavelength of 532 nm. The signal was detected using an InGaAs photomultiplier in combination with time correlated single-photon counting electronics at 10 K. The current density–voltage (*J-V*) measurement of the solar cell devices was performed using an AM1.5 solar simulator equipped under the standard test conditions (100 mW/cm^−2^, 25 °C) with a 300-W Xenon lamp (Model No. XES-100S1, SAN-EI, Japan). The active areas of all test cells were calibrated by an optical microscope (about 0.25 cm^2^). The EQE was measured by an Enlitech QER3011 system equipped with a 150-W xenon light source.

## Data Availability

All data used to evaluate the findings in the paper are present in the paper and/or the Supplementary Materials. Other relevant data are available from corresponding authors upon reasonable request.

## Supplementary Materials

Supplementary 1 Figs. S1 to S5 Computational method
