# Supplementary material for: Organic Passivation of Deep Defects in Cu(In,Ga)Se2 Film for Geometry-Simplified Compound Solar Cells
Source: Research (Wash D C). 2023 Mar 29;6:0084. doi: 10.34133/research.0084 (PMC10059681; doi:10.34133/research.0084)
Supplement: Supplementary 1 — Figs. S1 to S5 Computational method [file research.0084.f1.docx]

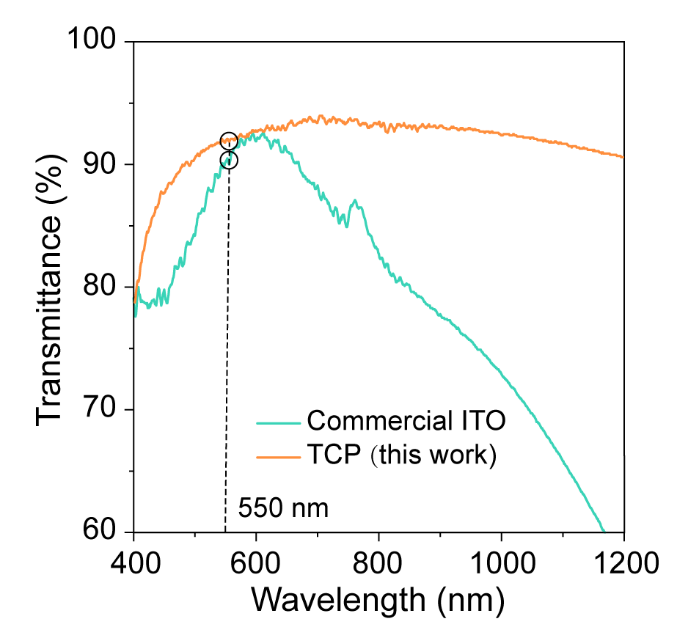


**Fig. S1 |** Transmittance of commercial ITO glass and TCP film in this work.

**
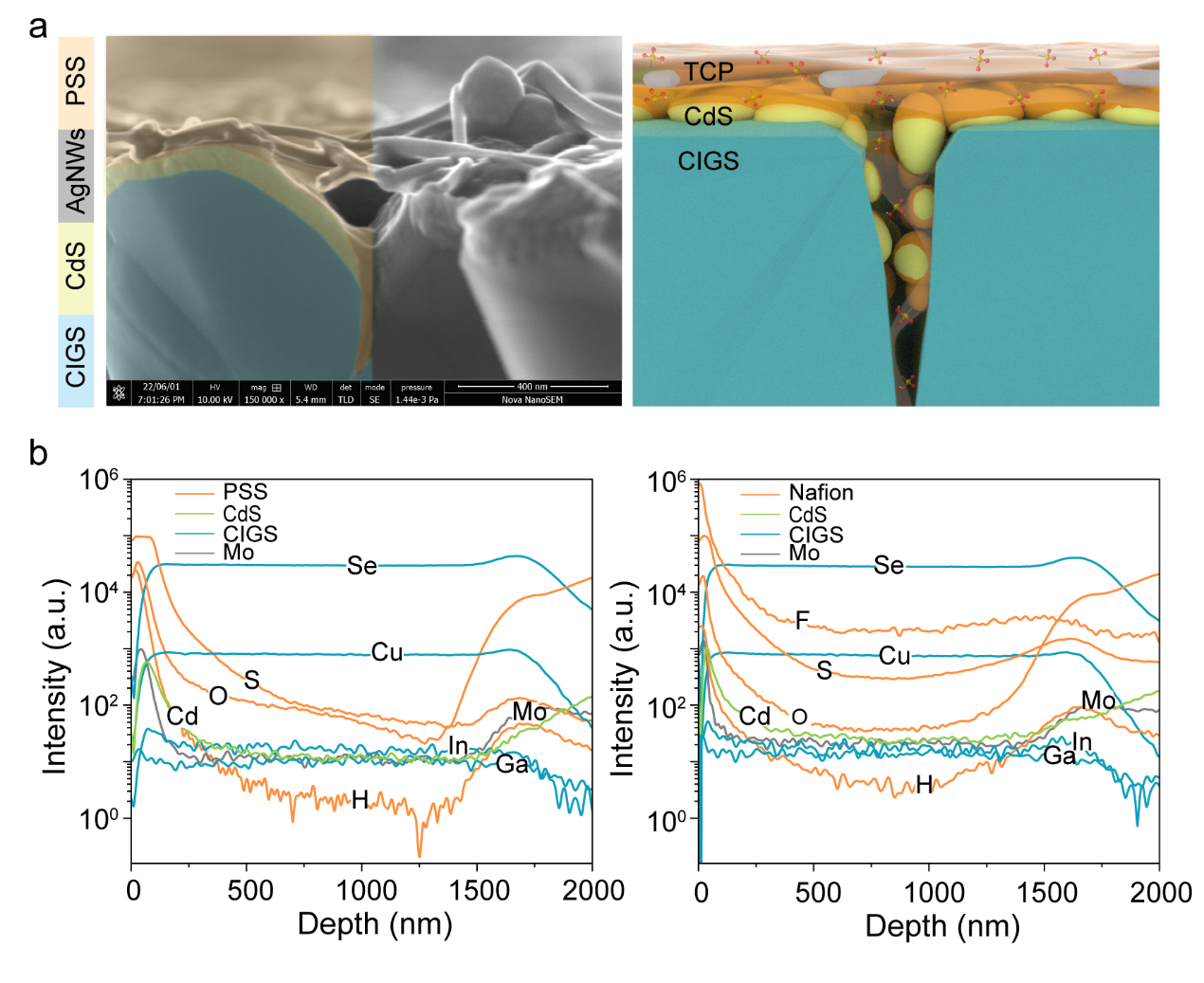
**

**Fig. S2 | a** SEM image (left) and schematic (right) of TCP/CdS/CIGS samples. As shown, the organic passivation agent penetrates into the CdS layer and reaches the CIGS interface. **b** Depth profiles in secondary ion mass spectrometry (SIMS) of PSS/CdS/CIGS and Nafion/ CdS/CIGS samples.

**
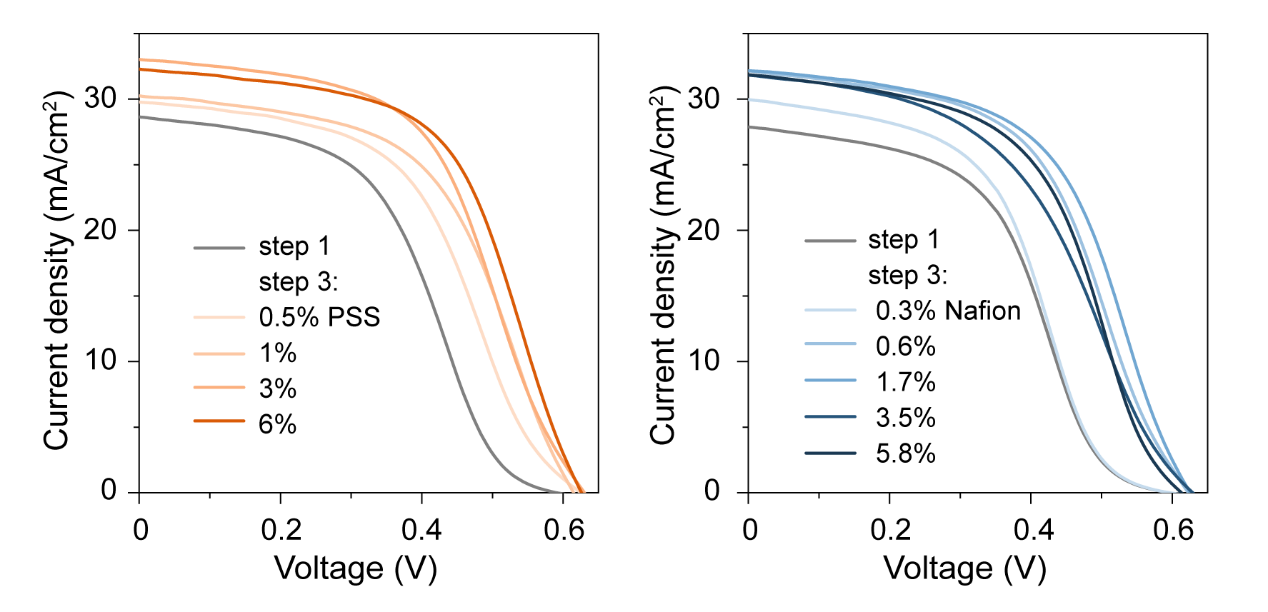
Fig. S3 |** The comparison of *J-V* curves of the architecture II solar cells before and after adding the PSS or Nafion passivation agent with different concentrations.


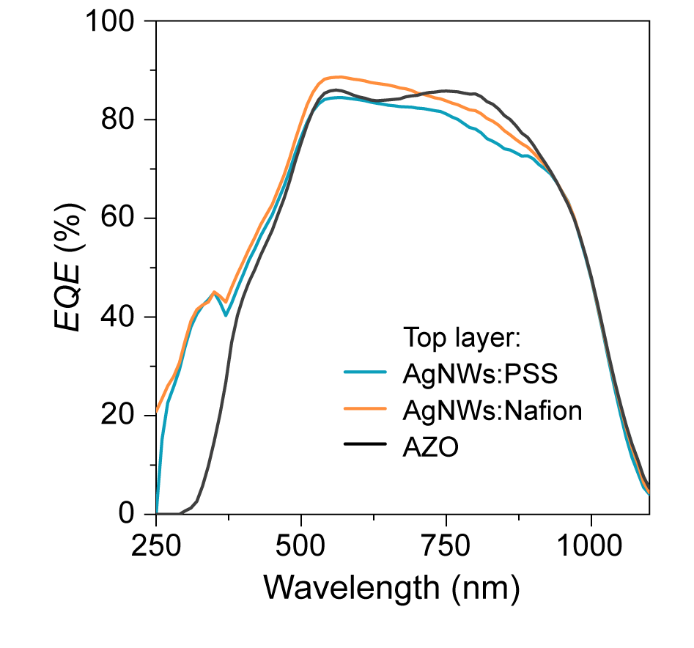


**Fig. S4 |** The comparison of external quantum efficiency (EQE) curves of the architecture II solar cells before and after adding the PSS or Nafion passivation, and AZO as a control.

**Fig. S5 |** Preparation process of three CIGS solar cells with different structures.


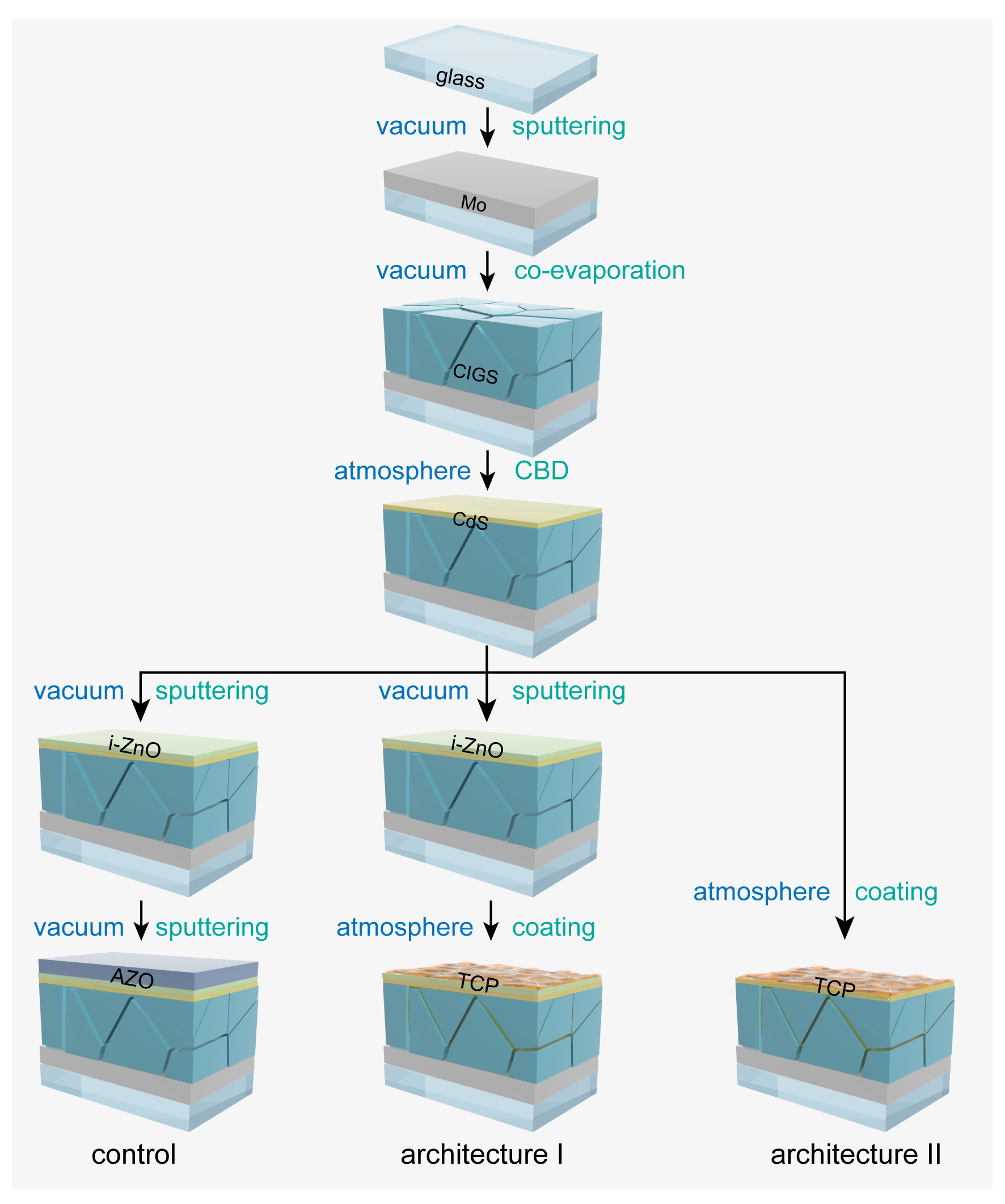


**Computational method**

Our calculations were performed by the Vienna Ab initio Simulation Package (VASP) ^1, 2^based on the density functional theory (DFT) using the projector augmented wave method (PAW)^3^ 0. A generalized gradient approximation (GGA) with the Perdew–Burke–Ernzerhof (PBE)^3^ function has been used for the exchange and correlation energy. The wave functions were expanded in plane waves with an energy cutoff of 500 eV and 3×4×1 Monkhorst-Pack k-point mesh^4^ was used for geometry optimization of all CIGS (112) surfaces and corresponding to a simple Nafion molecule adsorption system. A large vacuum layer of 30 Å was inserted to avoid interaction between adjacent slabs.

To describe the chemisorption of Nafion on the CIGS (112) surfaces, the adsorption energy *E*_a_ is defined as follows:

1. $E_{a}=E_{\mathrm{total}}- E_{\mathrm{CIGS}}-E_{\mathrm{Nafion}}$ (1)
2. where *E*_total_, *E*_CIGS_, and *E*_Nafion_ represent the total energy of the CIGS (112) surface–Nafion system, CIGS (112) surface, and Nafion energy, respectively. Herein, a simplified Nafion model (A simpler trifluoro methane sulfonic acid molecule) was used to represent the Nafion polymer to reduce the calculation time and decrease the computational complexities. According to the definition of the adsorption energy, a negative *E*_a_ indicates the stability of the adsorption structures. For all models, the top two layers of CIGS layers and Nafion molecule were allowed to relax, and the other two layers of the slab were fixed.

**References**

1. Kresse, G.; B, J. F. J. P. R., Efficient iterative schemes for ab initio total-energy calculations using a plane-wave basis set. **1996,** *54*.

2. Kresse, G.; Furthmüller, J., Efficiency of ab-initio total energy calculations for metals and semiconductors using a plane-wave basis set. *Computational Materials Science* **1996,** *6* (1), 15-50.

3. Perdew, J. P.; Burke, K.; Ernzerhof, M., Generalized Gradient Approximation Made Simple. *Physical Review Letters* **1996,** *77* (18), 3865-3868.

4. Monkhorst, H. J.; Pack, J. D., Special points for Brillouin-zone integrations. *Physical Review B* **1976,** *13* (12), 5188-5192.
